# GDF15 restrains myocardial ischemia-reperfusion injury through inhibiting GPX4 mediated ferroptosis

**DOI:** 10.18632/aging.205402

**Published:** 2024-01-10

**Authors:** Qingfeng Gao, Chao Li, Peiqi Zhong, Yunqiang Yu, Zhurong Luo, Hao Chen

**Affiliations:** 1 Department of Cardiovascular Medicine, The 900 Hospital of the Joint Service Support Force of the People’s Liberation Army of China, Fuzhou 350001, Fujian, China

**Keywords:** GDF1, myocardial ischemia-reperfusion injury, GPX4, ferroptosis

## Abstract

Background: Growth and differentiation factor 15 (GDF15) has been proved to regulate the process of Myocardial ischemia-reperfusion injury (MIRI), which is a serious complication of reperfusion therapy. The present study aimed to explore if GDF15 could regulate the MIRI-induced ferroptosis.

Method: MIRI animal model was established by ligating the left anterior descending coronary artery. Oxygen-glucose deprivation/reoxygenation (OGD/R) cell model was established to imitate MIRI *in vitro*. The indicators of ferroptosis including mitochondrial damage, GPX4, FACL4, XCT4, and oxidative stress markers were evaluated.

Results: Overexpression of GDF15 greatly inhibited MIRI, improved cardiac function, alleviated MIRI-induced ferroptosis. pc-DNA-GDF15 significantly inhibited the oxidative stress condition and inflammation response. The OGD/R-induced ferroptosis was also inhibited by pc-DNA-GDF15.

Conclusion: We proved that the MIRI-induced ferroptosis could by inhibited by pc-DNA-GDF15 through evaluating mitochondrial damage, MDA, GSH, and GSSG. Our research provides a new insight for the prevention and treatment of MIRI, and a new understanding for the mechanism of MIRI-induced ferroptosis.

## INTRODUCTION

Myocardial ischemia-reperfusion injury (MIRI) is a complex pathophysiological process that occurs when blood flow to the heart muscle is temporarily blocked and then restored. MIRI includes acute myocardial infarction (AMI), coronary artery bypass grafting (CABG), and percutaneous coronary intervention (PCI) [1–3]. During the ischemic phase, the heart muscle experiences oxygen and nutrient deprivation, leading to cell death and tissue damage [4, 5]. When blood flow is restored, a sudden influx of oxygen and inflammatory cells further exacerbates the injury, resulting in additional damage to the myocardium [6, 7]. Current therapies targeting MIRI mainly include reperfusion strategies, pharmacological interventions, cell therapy, and remote ischemic preconditioning (RIPC) [8, 9]. However, the prevention and treatment of MIRI are still a big challenge.

Recent research has revealed novel mechanisms involved in MIRI, providing new targets for potential therapeutic interventions. Growth and differentiation factor 15 (GDF15) belongs to the GDFs family and is a member of TGF-β superfamily [10]. A study has shown that GDF15 has an inhibitory effect on myocardial hypertrophy and may be a protective factor for the heart [11]. In addition, GDF15 knockout mice had larger infarct size and more cardiomyocyte apoptosis in the infarct border zone [12]. This indicates that endogenous GDF15 has an anti-myocardial injury effect. However, the underlying mechanism remains unclear.

Ferroptosis is a unique form of cell death that is distinct from apoptosis, necrosis, and autophagy. It is characterized by iron-dependent accumulation of lipid peroxides, leading to oxidative damage and cell death [13]. Emerging evidence suggests that ferroptosis, a novel form of regulated cell death characterized by iron-dependent lipid peroxidation, plays a crucial role in MIRI [14]. Dexmedetomidine inhibits MIRI-induced ferroptosis via AMPK/GSK-3β/Nrf2 axis [15]. Dapagliflozin alleviates myocardial ischemia/reperfusion injury by reducing ferroptosis via MAPK signalling inhibition [16]. If GDF15 could regulate MIRI through regulating ferroptosis has not been reported.

In the present study, the MIRI animal model was established, and oxygen-glucose deprivation/reoxygenation (OGD/R) was applied to imitate MIRI *in vitro*. Key ferroptosis indicators, including mitochondrial damage, malondialdehyde (MDA), glutathione (GSH), oxidized glutathione (GSSG), were measured. This research might provide a novel insight into the prevention and treatment of MIRI.

## MATERIALS AND METHODS

### MIRI animal experiment

A cohort of 24 male Sprague-Dawley rats (210–230 g) was obtained from Vital River Laboratory Animal Technology Co., Ltd., (Beijing, China) and randomly assigned to three groups (*n* = 8): Sham, MIRI, and MIRI+pcDNA-GDF15. The MIRI+pcDNA-GDF15 and MIRI groups received intravenous injections of pcDNA-GDF15 (4 × 10^12^ gc/kg) and the corresponding control vector, respectively. The vectors were injected 4 hours before operation once, and 2 hours after operation once. The dose of pcDNA-GDF15 and vectors were defined based on our pre-experiments and relevant publication [17]. Meanwhile, the rats in the Sham group were intravenously injected with the control vectors with same amount and time intervention. All experimental procedures were approved by the Institutional Ethics Committee for Laboratory Animal Care of the 900 Hospital of the Joint Service Support Force of the People’s Liberation Army of China.

To establish the MIRI animal model, rats were anesthetized using pentobarbital sodium (35 mg/kg, ip) and mechanically ventilated. Myocardial ischemia was induced by ligating the left anterior descending coronary artery with a 6-0 silk suture slipknot, as evidenced by ST-segment elevation on the electrocardiogram. After 40 minutes of ischemia, the slipknot was released to allow for myocardial reperfusion. In the sham group, rats underwent an identical procedure except for the ligation of the left anterior descending coronary artery. Echocardiographic measurements and blood sample collection were performed 24 hours after reperfusion. Subsequently, the rats were euthanized with an overdose of pentobarbital sodium (100 mg/kg, ip, #11715, Sigma, USA), and tissue samples were collected for further investigations.

### Cell culture and transfection

H9C2 cells (ATCC) were cultured in complete medium containing 10% FBS (#10099141C, Gibco, USA) at 37°C in an air-sealed chamber. Transfection of H9C2 with pcDNA-GDF15 or the control vector was performed with lipofectamine 2000 (#12566014, Invitrogen, USA). pcDNA-GDF15 and control vectors were designed and purchased from Shanghai GeneChem Co., Ltd., (Shanghai, China). pcDNA-GDF15 and control vectors were diluted with culture medium without serum to the final incubation concentration (50 nM).

### Establishment of OGD/R cell model

To establish the cell model of MIRI, OGD/R cell model cells were cultured in serum- and glucose-free complete medium and subjected to hypoxic conditions at 37°C for 6 hours. Subsequently, cells were maintained in normoxic conditions for 12 hours with complete medium containing 10% FBS (#10099141C, Gibco, USA) for reoxygenation.

### Hematoxylin-eosin (HE), massion, and sirius red staining

Cardiac tissues were fixed in 4% paraformaldehyde (#p0099, Beyotime, China) for 24 hours. Paraffin was used for embedding, and the tissues were cut into 6 μm-thick slices. HE staining was performed with hematoxylin and eosin staining. Masson staining and Sirius red staining were performed using a Masson’s Trichrome Stain kit and a Sirius Red Stain kit (#g1340, #g1472, Beijing Solarbio Science and Technology Co., Ltd., China), respectively, following the manufacturer’s instructions. The sections were then examined and photographed using an inverted optical microscope (Olympus Corporation, Japan).

### Echocardiographic measurement

Rats were administered pentobarbital sodium (45 mg/kg) for anesthesia and subsequently positioned supine to ensure immobilization. Subsequent evaluation employed a transthoracic echocardiography system (VisualSonics Inc., Canada) to assess two crucial cardiac function parameters: left ventricular fractional shortening (LVFS) and left ventricular ejection fraction (LVEF).

### Immunofluorescence and immunohistochemistry (IHC) staining

Tissues were prepared as described in the part 2.4. Tissue sections were subjected to fixation in 4% paraformaldehyde for a duration of 30 minutes, succeeded by a tripartite rinse with phosphate-buffered saline (PBS, #p1010, Beijing Solarbio Science and Technology Co., Ltd.). Upon subsequent immersion in a blocking buffer designed for immunohistochemical staining (Beyotime, China) at room temperature, a preparatory period of 15 minutes ensued. The sections were subsequently subjected to an overnight incubation at 37°C with primary antibody (dilution 1:100; Abcam). A subsequent step involved an hour-long incubation with a second antibody (dilution 1:200; Beyotime, China) at room temperature. Visualization and documentation of the samples were performed using a confocal laser-scanning microscope (Leica Microsystems GmbH). The quantification of microvessel fluorescence density was conducted using ImageJ software.

### Western blot analysis

Tissues and cells underwent lysis in RIPA buffer (#P0013B, Beyotime, China), supplemented with 1% protease inhibitor. The protein content was quantified using the BCA assay kit (#P0009, Beyotime, China). Thereafter, same amount of protein was subjected to separation by 10% SDS-PAGE and subsequently transferred onto polyvinylidene fluoride (PVDF) membranes (#IPVH00010, Millipore, USA). The membranes were incubated in a 5% skim milk solution at ambient temperature for 2 hours. Subsequent immunoblotting was accomplished by overnight incubation at 4°C with primary antibodies. The membranes were exposed to horseradish peroxidase-conjugated goat anti-rabbit IgG (Abcam, UK) at ambient temperature for 2 hours. Visualization of protein bands was achieved through utilization of an enhanced chemiluminescence kit (#32132, Thermo Fisher Scientific, USA) on a chemiluminescence imaging system (Bio-Rad Laboratories, Inc., USA). Quantification of protein band intensity was conducted utilizing ImageJ software (Bio-Rad Laboratories, Inc., USA). The used antibodies are listed below: GPX4 (#ab125066, Abcam, UK); FACL4 (ab155282, Abcam, UK); XCT4 (#26864-1-AP, Thermo Ficher Scientific, USA); GAPDH (#4366, Abcam, UK).

### Measurement of MDA, GSH, GSSG, IL-1β, TGFβ and IL-6

For acquisition of serum samples, blood was collected from the carotid artery of rats subjected to pentobarbital sodium anesthesia (45 mg/kg). The samples were maintained at room temperature for a 2-hour interval prior to centrifugation at 1,000 × g for 15 minutes. Levels of SOD (#BC0170), MDA (#BC0025), GSH (#BC1175), IL-1β (#SEKR0002), TGFβ (#SEKR0012) and IL-6 (#SEKR005) were determined utilizing appropriate assay kits (Beijing Solarbio Science and Technology Co., Ltd.), as per the manufacturer’s guidelines.

### CCK8 assay

Cells were seeded into 96-well plates at a density of 2 × 10^3^ cells per well and subjected to OGD/R treatment. Post-treatment, cells were rinsed with PBS and then exposed to 0.5 mg/ml CCK8 reagent (#CA1210, Beijing Solarbio Science and Technology Co., Ltd.) at 37°C for 4 hours. The absorbance was measured at a wavelength of 570 nm using a microplate reader.

### Wound healing assay

The wound healing assay was conducted to assess the migratory capacity of cells. Initially, cells were seeded at a density of 4 × 10^5^ cells per well in a six-well plate and maintained in serum-deprived medium until reaching approximately 90% confluence. Subsequently, the confluent cell monolayer was subjected to controlled mechanical injury by gently scraping with a sterile 1 mL pipette tip. Following injury, the cells were rinsed twice with sterile PBS. The remaining adherent cells were then subjected to OGD/R treatment and cultured under serum-deprived conditions for a duration of 48 hours. Photomicrographs capturing the wound area were acquired at both the initial time point (0 hours) and after the 24-hour incubation period, utilizing an inverted optical microscope manufactured by Olympus Corporation.

### Transwell assay

To evaluate the invasive potential of cells, a transwell assay was employed cells at a quantity of 4 × 10^5^ cells were introduced into the upper chambers of transwell inserts featuring an 8 μm pore size (Millipore, USA). The cells were then cultivated in serum-deprived medium, while the lower chambers were supplied with complete medium supplemented with 15% fetal bovine serum (FBS). Following exposure to the OGD/R for a duration of 48 hours, the cells that had migrated to the lower surface of the upper chamber were fixed using a solution of 4% paraformaldehyde for 30 minutes. Following fixation, these cells were stained with 0.1% crystal violet (#C0121, Beyotime, China) for 15 minutes. Post-staining, the cells were washed twice with PBS, and the migrated cells were visualized and quantified through utilization of an inverted optical microscope manufactured by Olympus Corporation. The quantification process involved tallying the number of migrated cells, which facilitated subsequent data analysis.

### Flow cytometry

Cellular apoptotic events were analysed through the utilization of flow cytometry. The cell population obtained post-digestion was subjected to centrifugation at 2000 g for a duration of 15 minutes. The resulting supernatant was removed, and the cells were treated with a cell apoptosis kit containing both propidium iodide and Annexin V-FITC (#C1062S, Beyotime, China). This incubation occurred under light-protected conditions for a period of 15 minutes. Subsequently, flow cytometry was performed to analyse and quantify the extent of cellular apoptosis.

### Statistical analysis

Statistical analysis of the acquired data was executed in conformity with established methodologies. The presentation of data adhered to the convention of mean values supplemented with standard deviation. The statistical discrepancies were assessed via one-way analysis of variance (ANOVA), followed by the Bonferroni’s post hoc test for pairwise comparisons. The software employed for statistical analysis was SPSS (version 22.0, IBM Corp.). A significance threshold of *P* < 0.05 was applied to demarcate statistically meaningful differences within the experimental results.

### Availability of data and materials

The datasets used and/or analyzed are available from the corresponding authors on reasonable request.

## RESULTS

### Overexpression of GDF15 greatly inhibited MIRI and improved cardiac function

**Figure 1:**
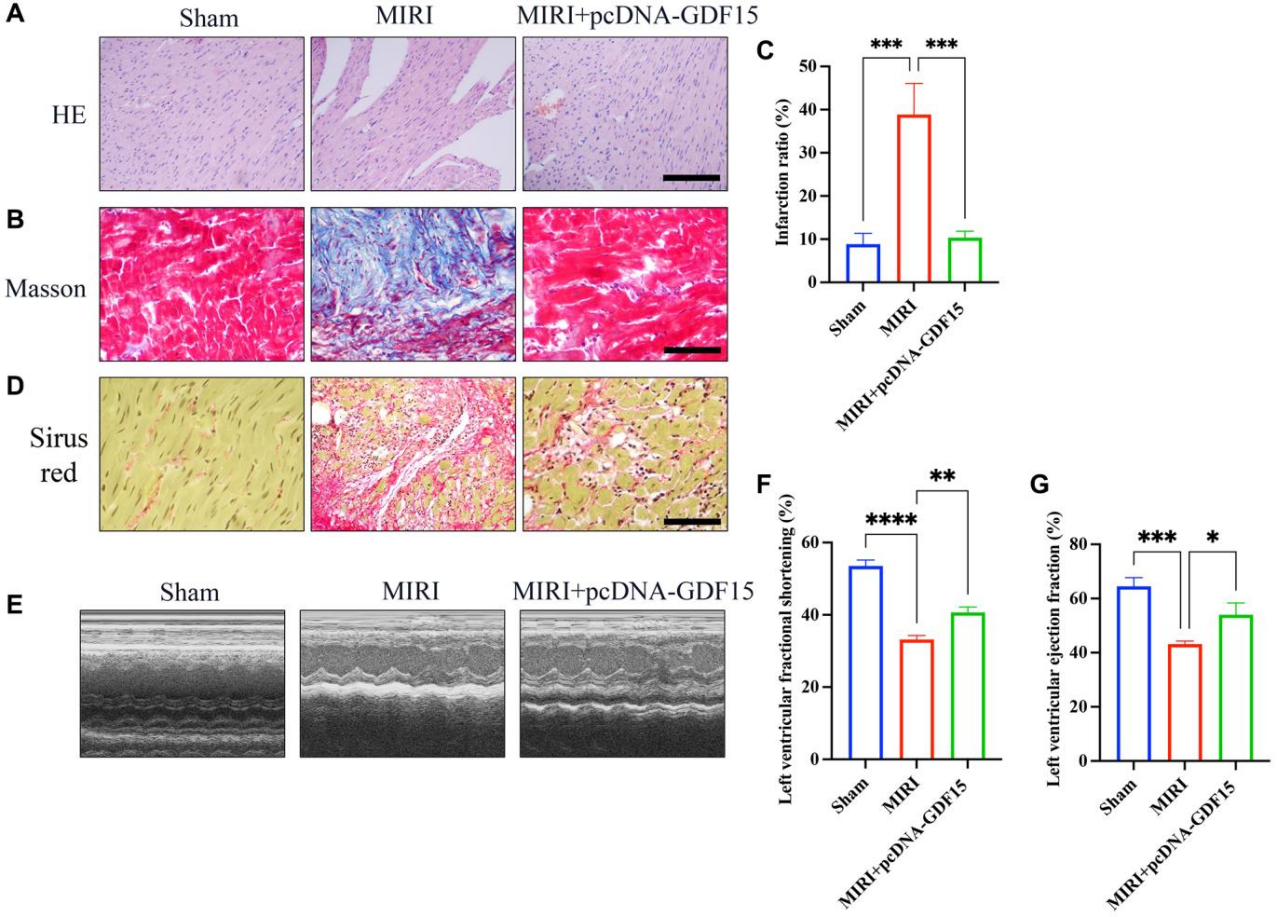
**Overexpression of GDF15 greatly inhibited MIRI and improved cardiac function.** (**A**) HE staining of cardiac tissues in different groups (bar: 200 μm, magnification: 100×); (**B**, **C**) Masson staining of cardiac tissues in different groups and infarction ration analysis (bar: 200 μm, magnification: 100×); (**D**) Sirius red staining of cardiac tissues in different groups (bar: 200 μm, magnification: 100×). (**E**–**G**) Echocardiographic measurement, and analysis of LVFS and LVEF. ^*^P < 0.05, ^**^P < 0.01, ^***^P < 0.001.

The results of HE staining demonstrated that myocardial cells in sham rats were well-arranged and possessed intact muscle fibres, while myocardial cells became irregularly arranged and obvious necrosis was observed in group MIRI. However, treatment with pcDNA-GDF15 greatly ameliorated histopathological changes (Figure 1A). In addition, obvious collagen deposition in MIRI was evaluated with Masson staining and Sirius red staining, which was suppressed by pcDNA-GDF15 (Figure 1B–1D). The finds also revealed that LVFS and LVEF in MIRI rats were significantly restrained compared with sham rats, and these decreases were significantly reversed by pcDNA-GDF15 (Figure 1E–1G).

### The ferroptosis induced by MIRI was remarkably alleviated by pc-DNA-GDF15

**Figure 2:**
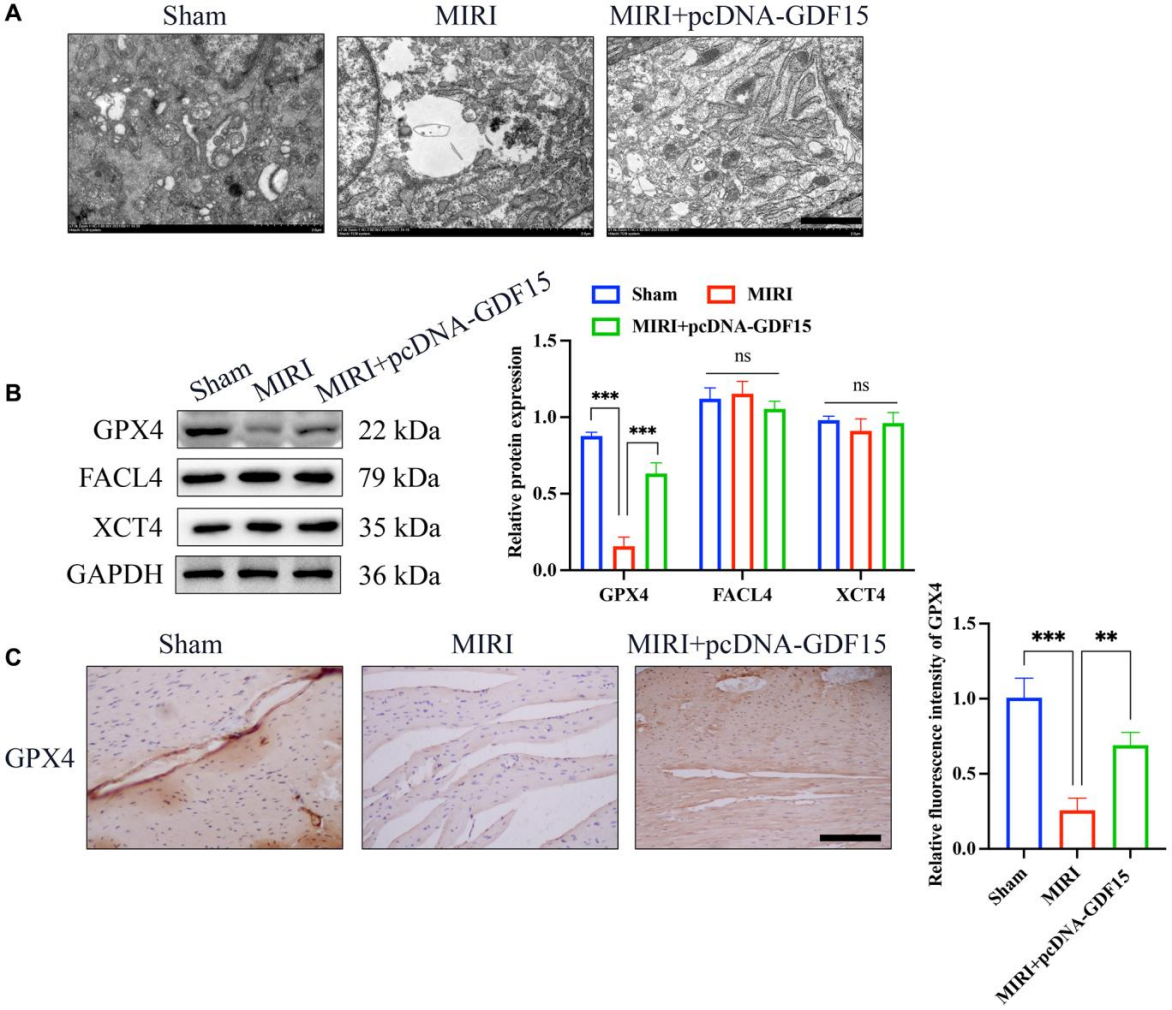
**The ferroptosis induced by MIRI was remarkably alleviated by pc-DNA-GDF15.** (**A**) Detection of mitochondrial damage with TEM (bar: 100 nm); (**B**) Relative protein expression levels of GPX4, FACL4, and XCT4 in heart tissues. (**C**) IHC staining of GPX4 in the heart tissues (bar: 200 μm, magnification: 100×). ^*^P < 0.05, ^**^P < 0.01, ^***^P < 0.001.

Mitochondrial damage is one of the markers of ferroptosis process. We found that the MIRI group had reduced mitochondrial size and membrane damage, but pcDNA-GDF15 treatment significantly inhibited mitochondrial damage (Figure 2A). Glutathione peroxidase 4 (GPX4), FACL4, and XCT4 are key regulators of ferroptosis. We found that the level of GPX4 was greatly inhibited in the group MIRI, but the levels of FACL4 and XCT4 remained unchanged. Meanwhile, the decreased GPX4 was increased after overexpression of GDF15 (Figure 2B, 2C), which indicate that GDF15 might regulate the ferroptosis process through targeting GPX4.

### pc-DNA-GDF15 significantly inhibited the oxidative stress condition and inflammation response

**Figure 3:**
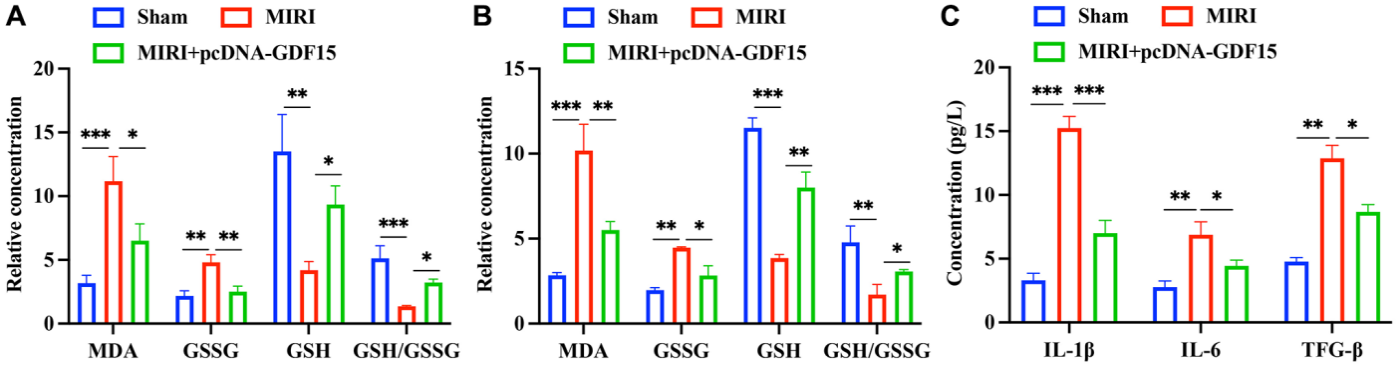
**pc-DNA-GDF15 significantly inhibited the oxidative stress condition and inflammation response.** (**A**, **B**) Detection of oxidative stress biomarkers including MDA, GSSG, GSH in the serum and myocardial tissues. (**C**) Measurement of the serum levels of IL-1β, TGF-β, and IL-6. ^*^P < 0.05, ^**^P < 0.01, ^***^P < 0.001.

Oxidative stress biomarkers in the serum and myocardial tissues were subsequently measured. The levels of MDA and GSSG in the group MIRI were remarkably increased, but decreased after pcDNA-GDF15 treatment (Figure 3A, 3B). Meanwhile, the decreased GSH and GSH/GSSG were greatly promoted in the group MIRI+pcDNA-GDF15 (Figure 3A, 3B). In addition, the expression of IL-1β, TGF-β, and IL-6 in MIRI rats were significantly increased compared with that in sham animals, and pcDNA-GDF15 significantly reversed these increases in MIRI rats (Figure 3C). These results indicate that pcDNA-GDF15 significantly reduced oxidative stress and inflammation response in MIRI rats.

### pc-DNA-GDF15 significantly inhibited the ferroptosis in OGD/R-treated H9C2 cells

**Figure 4:**
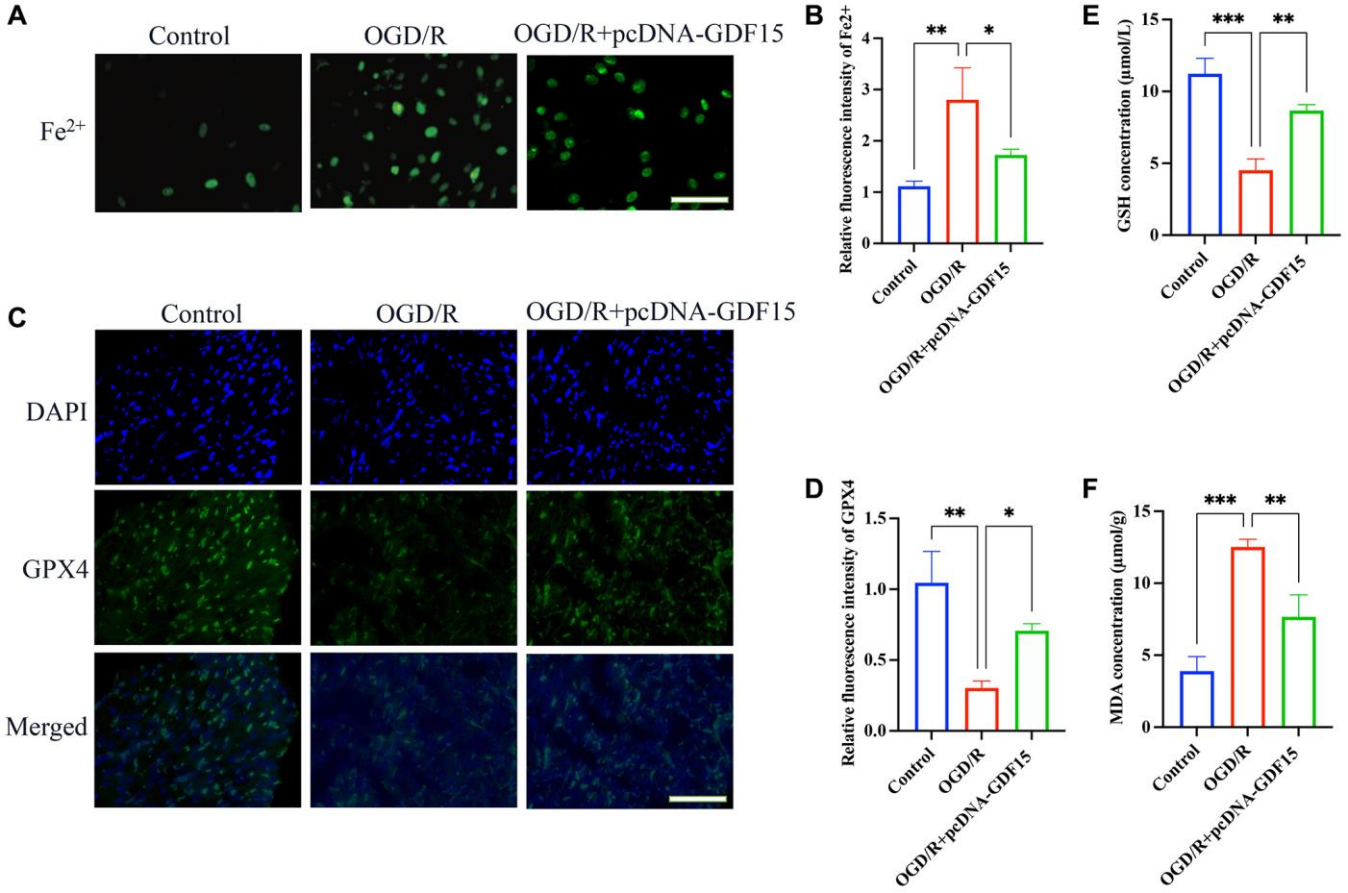
**pc-DNA-GDF15 significantly inhibited the ferroptosis in OGD/R-treated H9C2 cells.** (**A**, **B**) The Fe^2+^ level in cells were validated (bar: 100 μm, magnification: 100×); (**C**, **D**) The relative expression intensity of GPX4 in the cells were evaluated (bar: 200 μm, magnification: 40×); (**E**, **F**) The levels of GSH and MDA in the cells were measured. ^*^P < 0.05, ^**^P < 0.01, ^***^P < 0.001.

The influence of GDF15 on MIRI-induced ferroptosis was also validated *in vitro* using OGD/R cell model. pc-DNA-GDF15 significantly decreased the intracellular Fe^2+^ production (Figure 4A, 4B) and increased GPX4 expression (Figure 4C, 4D) in OGD/R-treated cells compared with group OGD/R. Meanwhile, the remarkable decreased GSH (Figure 4E) and increased MDA (Figure 4F) caused by OGD/R were greatly reversed after overexpression of GDF15.

### pc-DNA-GDF15 significantly inhibited the ferroptosis in OGD/R-treated H9C2 cells

**Figure 5:**
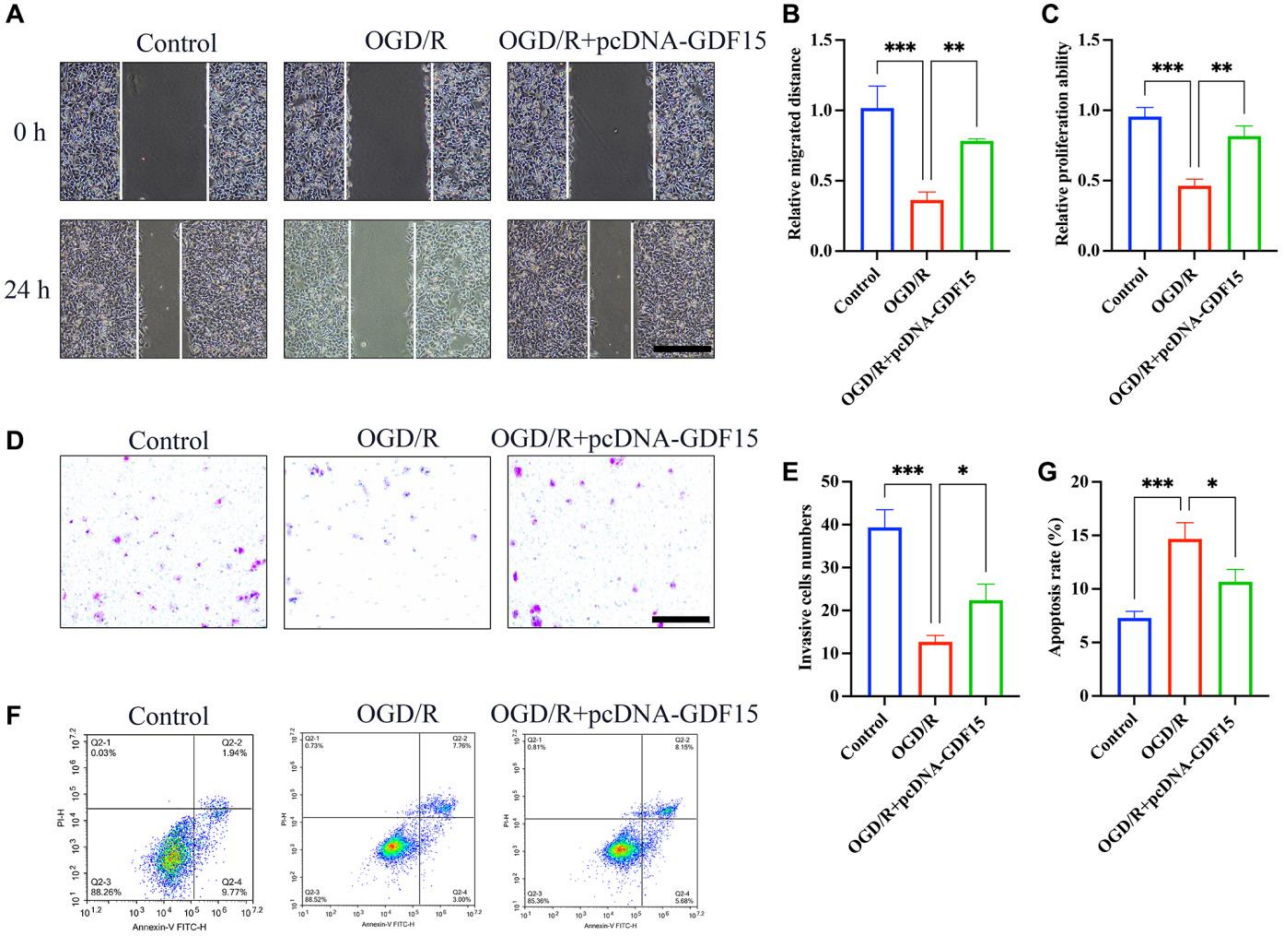
**pc-DNA-GDF15 significantly inhibited the ferroptosis in OGD/R-treated H9C2 cells.** (**A**, **B**) Measurement of cell migration with wound healing assay (bar: 400 μm, magnification: 10×); (**C**) Detection of cell proliferation with CCK8; (**D**, **E**) Measurement of cell invasion with Transwell assay (bar: 100 μm, magnification: 100×); (**F**, **G**) Measurement of cell migration with flow cytometry. ^*^P < 0.05, ^**^P < 0.01, ^***^P < 0.001.

pc-DNA-GDF15 significantly enhanced the migration (Figure 5A, 5B), proliferation (Figure 5C) and invasion (Figure 5D, 5E) of OGD/R-treated H9C2 cells compared with signal treatment with OGD/R. Meanwhile, the great increased cell apoptosis in OGD/R-treated H9C2 cells was markedly decreased by treatment with pc-DNA-GDF15 (Figure 5F–5G). Based on this result, we confirmed that the transfection of pc-DNA-GDF15 in H9C2 cells could affect the OGD/R-induced ferroptosis.

## DISCUSSION

AMI is a series of acute cardiovascular syndromes caused by blockage or severe stenosis of coronary artery blood flow due to various factors, resulting in myocardial hypoxia, ischemia, and hypoxia necrosis of myocardial cells [18]. Revascularization greatly reduces the size of myocardial infarction and improves patient prognosis [19]. However, myocardial ischemia-reperfusion injury further aggravates the damage of myocardial cells, causes apoptosis of myocardial cells, and leads to a decrease in the number of viable myocardium [20].

The key regulators of ferroptosis include GPX4, the iron-dependent enzyme, and lipoxygenase (LOX) [21]. Dysregulation of these components can lead to the accumulation of lipid peroxides and ultimately trigger ferroptosis [1]. During ischemia, the disruption of cellular homeostasis results in the accumulation of reactive oxygen species (ROS) and iron, which promote lipid peroxidation [3]. Upon reperfusion, the sudden influx of oxygen and iron exacerbates lipid peroxidation, leading to ferroptotic cell death [7]. Furthermore, the downregulation of GPX4, a key enzyme that protects against lipid peroxidation, has been observed in MIRI, indicating its role in the regulation of ferroptosis in this context [22, 23]. We found that the decreased GPX4 expression caused by MIRI and OGD/R was greatly increased by pcDNA-GDF15, which might explain the function mechanism of GDF15 in regulating ferroptosis.

FACL4, also known as ACSL4 (Acyl-CoA Synthetase Long-Chain Family Member 4), is a key enzyme involved in lipid metabolism and lipid peroxidation, which plays a crucial role in ferroptosis [24]. FACL4 is known to be upregulated in response to oxidative stress and is one of the key regulators of lipid metabolism in ferroptosis [25]. XCT4 plays a critical role in the regulation of intracellular cysteine levels and redox balance, and it is crucial for the synthesis of GSH [26]. We demonstrated that the levels of both FACL4 and XCT4 remained unchanged after MIRI and pcDNA-GDF15 treatment, indicating that they are not linked with MIRI-induced ferroptosis. Meanwhile, these findings suggest that GDF15 does not affect MIRI-induced ferroptosis through FACL4 and XCT4.

MDA is a well-characterized and abundant end product of lipid peroxidation. During ferroptosis, the excessive accumulation of lipid hydroperoxides leads to the generation of MDA. Measuring MDA levels can therefore serve as a direct indicator of lipid peroxidation and provides a reliable read-out for ferroptosis. GSH is a tripeptide that plays a crucial role in maintaining cellular redox homeostasis. It acts as a key antioxidant by scavenging reactive oxygen species (ROS) and directly reducing lipid hydroperoxides. During ferroptosis, the depletion of GSH due to excessive oxidative stress is considered a hallmark [27]. Therefore, detection of MDA and GSH provide valuable information about the cellular antioxidant capacity and the occurrence of ferroptosis.

Myocardial cell apoptosis after OGD/R is the key to the decline of myocardial reserve after MIRI. The occurrence of apoptosis is closely related to energy metabolism, death signal pathway, endoplasmic reticulum stress, and mitochondrial dysfunction. Therefore, understanding the transcriptional molecular regulation mechanism of myocardial cell apoptosis during the development of MIRI is expected to find key regulatory molecules. In this research, we found that overexpression of GDF15 greatly inhibited the cell apoptosis after OGD/R treatment. There are several limitations in this research. (1) The further targeting signaling pathway of GDF15 remains unclear. (2) How GDF regulate the ferroptosis during MIRI was not clarified.

## CONCLUSION

We demonstrated that the MIRI could further cause ferroptosis through evaluating mitochondrial damage, MDA, GSH, and GSSG. The MIRI-induced ferroptosis was greatly suppressed after overexpression of GDF15 through GPX4. Our research provides a novel thought for the prevention and treatment of MIRI, and a new understanding for the mechanism of MIRI-induced ferroptosis.
